# Development of Solid-Phase Microextraction with Carbon Dot-Functionalized Hollow Fiber Membrane for the Analysis of Perfluoroalkyl Carboxylates in Aqueous Samples

**DOI:** 10.3390/molecules31081255

**Published:** 2026-04-10

**Authors:** Chaoyan Lou, Shaojie Pan, Kaidi Zhang, Xiaolin Yu, Shijie Wei, Yang Lu, Kai Zhang, Yan Zhu

**Affiliations:** 1 College of Quality and Standardization, China Jiliang University, Hangzhou 310018, China; louchaoyan@cjlu.edu.cn (C.L.); 18868730104@163.com (S.P.);; 2 Zhejiang Institute of Quality Sciences, Hangzhou 310018, China; 3 Ningbo Key Laboratory of Agricultural Germplasm Resources Mining and Environmental Regulation, College of Science and Technology, Ningbo University, Ningbo 315300, China; zhangkai1@nbu.edu.cn; 4 Department of Chemistry, Zhejiang University, Hangzhou 310028, China

**Keywords:** nitrogen-doped carbon dot-functionalized hollow fiber membrane (NCDs@HFM), perfluoroalkyl carboxylates (PFCAs), solid-phase microextraction mode (SPME), liquid chromatography–tandem mass spectrometry (LC-MS/MS), composite membrane, emerging pollutants

## Abstract

Due to the ultra-trace concentrations of perfluoroalkyl compounds (PFCs) existing in environmental aqueous matrices, it is imperative to develop sensitive and high-enrichment-efficiency approaches for the determination of these emerging pollutants. In this study, a nitrogen-doped carbon dot-functionalized hollow fiber membrane (NCDs@HFM) was fabricated and employed in solid-phase microextraction (SPME) mode for the simultaneous identification of eight perfluoroalkyl carboxylates (PFCAs). The NCDs@HFM offers several advantages, including multiple active binding sites, chemical durability, a large specific surface area and environmental compatibility. Owing to these properties, the NCDs@HFM-based SPME demonstrated high extraction efficiency for PFCAs, where enrichment factors for target molecules could reach 35–61 fold under the optimum conditions. This established method was then integrated with liquid chromatography–tandem mass spectrometry (LC-MS/MS) for the qualitative and quantitative analysis of eight representative PFCAs in drinking and environmental water samples. The limits of detection (LODs, S/N = 3) and quantitation (LOQs, S/N = 10) of the method were at the scale of 0.0018–0.015 μg/L and 0.006–0.050 μg/L, respectively. This proposed method exhibited good precision, with RSDs below 13.2% and satisfactory accuracy, with recoveries ranging from 70.6% to 122.5%. The developed method was successfully applied in the identification of eight typical PFCAs in drinking and environmental water samples. This method exhibits several merits, including low cost, high sensitivity, good reliability and reusability, representing a promising alternative for measuring trace levels of PFCAs in aqueous matrices.

## 1. Introduction

Perfluoroalkyl compounds (PFCs) are a family of synthetic chemicals in which all hydrogen atoms in the alkyl chain are substituted with fluorine atoms. The carbon–fluorine bond, with a bond energy of C-F of 488 kJ/mol, is one of the strongest covalent bonds in organic chemistry, endowing PFCs with exceptional thermal stability, chemical resistance, and both hydrophobic and oleophobic characteristics [1,2]. These distinctive properties have led to their widespread use in numerous industrial and commercial applications, including non-stick cookware, textiles, upholstery, food packaging and electroplating. However, increasing toxicological evidence has unequivocally highlighted the environmental risks and bioaccumulation potential associated with PFCs [3,4,5]. Due to their robust chemical stability and amphiphilic properties, PFCs are resistant to degradation in the environment and prone to long-range transport, finally bioaccumulating in living organisms [6,7,8,9].

PFCs are generally partitioned into two principal categories, perfluoroalkyl carboxylates (PFCAs) and perfluoroalkyl sulfonates (PFSAs) [10], which predominantly exist as anionic species (pKa < 2) in aqueous matrices [11]. PFCAs are characterized by fully carbon–fluorine bonds terminated by a carboxylic acid group. Compared to PFSAs, PFCAs often exhibit greater environmental mobility due to their relatively higher water solubility and lower molecular weight, which facilitate wider dispersion in aquatic systems. Alarmingly, extensive epidemiological investigations and toxicological assessments have established compelling associations between chronic PFCAs exposure and multi-organ health effects, including hepatic dysfunction, immune suppression, endocrine disruption, and developmental abnormalities [12,13]. The length of the carbon chain significantly influences the physical and chemical properties of PFCAs. Generally, long-chain PFCAs exhibit enhanced environmental persistence compared to short-chain analogs and exhibit higher bioaccumulation in organisms [14]. As a consequence, long-chain PFCAs (e.g., perfluorooctanoic acid (PFOA)) have been officially designated as persistent organic pollutants (POPs) under the Stockholm Convention since 2009. Moreover, the International Agency for Research on Cancer (IARC, 2023) has classified PFOA as “possibly carcinogenic to humans”. On this basis, Global regulatory authorities, including the U.S. Environmental Protection Agency (EPA, 2024) and the European Union’s REACH Regulation (EC No. 1907/2006), have issued health advisories to limit human exposure. For instance, the EPA has set a health advisory level for PFOA and its salts in drinking water at 4 μg/L (individually or combined) [15]. Despite these regulations, monitoring PFCAs remains challenging, attributed to their ultra-trace concentrations in environmental matrices. Accordingly, the development of highly efficient and sensitive analytical methods for the detection and quantification of PFCAs is of great importance.

Several analytical methodologies for perfluoroalkyl compounds have been reported in previous studies, such as liquid chromatography (LC) [16], gas chromatography (GC) [17] and their hyphenated mass spectrometry techniques [18,19,20,21]. Among these techniques, liquid chromatography tandem mass spectrometry (LC-MS/MS) has become the preferred method due to its high accuracy, sensitivity and the ability to analyze molecules without derivatization [22]. However, to eliminate matrix interferences of complicated samples as well as to preconcentrate target analytes, appropriate sample preparation methods remain essential prior to chromatographic separations [23]. At present, a variety of sample preparation methods [24] have been investigated including solid phase extraction (SPE) [25,26], liquid–liquid extraction (LLE) [27], solid-phase microextraction (SPME) [28], dispersive solid phase extraction (d-SPE), magnetic solid phase extraction (M-SPE) [29] and QuEChERS method [20,30]. Among the above-mentioned methods, SPME has demonstrated significant potential for enriching PFCAs owing to its operational simplicity, low organic solvent consumption, and high enrichment factors. In this perspective, SPME has been considered as the recommended sample pretreatment method in several testing standards. For instance, ISO 21675:2019 [31] specifies an SPME method based on a copolymer-based ion exchange membrane for extracting perfluorinated compounds from water. This method exhibits acceptable extraction efficiency for short-chain perfluoroalkyl molecules [31] while the recovery of long-chain perfluoroalkyl molecules is not quite satisfactory due to the low affinity of the membrane for long-chain perfluoroalkyl compounds. Given these challenges, there is still a pressing and continuous demand to make some advancements in the SPME methodology.

In SPME methodology, the hollow fiber membrane is the most critical factor determining the extraction capacity and adsorption selectivity. In comparison with conventional fibrous materials, composite membrane materials with functionalized sites are the preferred choice, especially for the trace-level compounds enrichment application scenarios. The concept of carbon dots was introduced to prepare a composite membrane in this work. Carbon dots are a zero-dimensional material that can be incorporated with a pristine membrane to form the functionalized surface layer [32]. Specifically, core–shell structured nitrogen-doped carbon dots (NCDs) were designed and synthesized, featuring abundant reactive groups on their surface to endow the membrane with enhanced adsorption efficiency. The as-prepared NCDs exhibit good adsorption performance due to their ultra-small size (2–20 nm), rich chemical groups (e.g., amino, carboxyl, and hydroxyl groups) and robust physical and chemical properties. These properties confer composite membranes superior adsorption and extraction abilities, enabling them to enrich PFCAs in water samples. As a consequence, a modified SPME method based on nitrogen-doped carbon quantum dots functionalized hollow fiber membrane (NCDs@HFM) was creatively proposed and successfully used together with LC-MS/MS for the PFCAs analysis in this work. This method was optimized and validated to provide an innovative approach for the rapid and high-throughput analysis of PFCAs at low concentration levels.

## 2. Results and Discussion

### 2.1. LC-MS/MS Method Development

The chromatographic conditions were systematically optimized to achieve satisfactory separation of all eight target PFCAs. Firstly, different organic solvents, including methanol and acetonitrile, were tested. Methanol was ultimately selected as it provided higher signal intensities and better peak shapes for the target PFCAs. For the aqueous phase, ammonium acetate at a concentration of 5 mmol/L was chosen as it effectively sharpens the peaks. A gradient elution program (Section 3.4) was established to achieve better peak shape, higher resolution and shorter analysis time. The chromatogram of the PFCAs’ standard is depicted in Figure S1.

As for mass spectrometry, both positive and negative electrospray ionization modes could be used. In this study, the negative ESI mode was selected for the mass spectrometric acquisition as it yielded significantly higher signal intensity and better stability for PFCAs. The qualitative and quantitative analyses were conducted in the multiple reaction monitoring (MRM) mode. The target analyte was identified according to retention time and precursor ion transition. The capillary and nozzle voltages were optimized to 3500 V and 2000 V to achieve satisfactory precursor ion signals. Other parameters (drying gas temperature: 200 °C; drying gas flow rate: 11 L/min; sheath gas temperature: 150 °C; sheath gas flow rate: 8 L/min; nebulizer pressure: 35 psi) were maintained as described.

### 2.2. Optimization of NCDs@HFM SPME Conditions

To enhance the extraction performance of the proposed NCDs@HFM SPME method, several operating parameters, including the types of HFM, adsorption conditions and desorption conditions, were investigated.

#### 2.2.1. Optimization of Hollow Fiber Membrane

**Figure 1:**
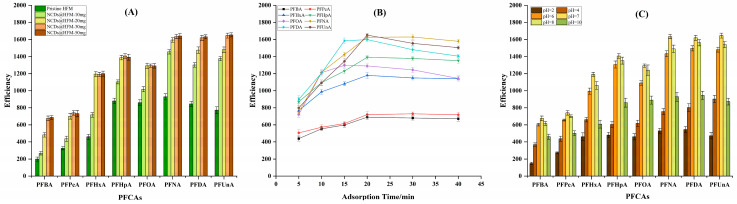
Optimization of the NCDs@HFM-SPME conditions for the extraction of PFCAs. The effects of (**A**) NCDs loading amount (0, 10, 20, 30 and 50 mg) on the hollow fiber membrane, (**B**) adsorption time (from 0 to 40 min), and (**C**) sample solution pH (from 2 to 10) on the extraction efficiency are evaluated. Optimization experiments were performed using a standard solution containing the eight target PFCAs, each at a concentration of 1.0 μg/L. Error bars represent the standard deviations of triplicate measurements.

In SPME methodology, the extraction performance mainly depends on the adsorption capacity of the hollow fiber membrane. To optimize this critical component, HFMs were functionalized with varying amounts of nitrogen-doped carbon dots (0, 10, 20, 30 and 50 mg). The extraction performance of NCDs@ HFM was far better than bare HFM. This is probably due to functional groups on the shell of NCDs, which have a strong electrostatic interaction with anionic carboxylate groups of the PFCAs. As shown in Figure 1A, the extraction efficiency for all target PFCAs increased with NCDs loading from 10 mg to 30 mg, reaching an adsorption equilibrium at the amount of 30 mg NCDs. There were no significant changes in extraction efficiency when the amount of NCDs increased continuously. Hence, a hollow fiber membrane incorporated with 30 mg NCDs was applied in the SPME procedure in the following experiments.

#### 2.2.2. Optimization of Adsorption Conditions

To obtain the satisfactory adsorption efficiency of PFCAs by the proposed method, several factors, including sample solution pH and adsorption time, were investigated. As an equilibrium-based process, the extraction efficiency in SPME is highly dependent on adsorption time. In this study, the effect of adsorption time was evaluated over a range of 0 to 40 min. As depicted in Figure 1B, the extraction efficiency of each target analyte increased for the first 20 min; when the time was further prolonged, the extraction efficiency stabilized for short-chain PFCAs but decreased for long-chain PFCAs. This decline for long-chain compounds may be attributed to their gradual desorption or competitive adsorption dynamics over extended periods. Based on the experimental results, an adsorption time of 20 min was selected for the NCDs@HFM-SPME method to ensure robust extraction across all target analytes.

Moreover, the sample solution pH is another parameter that will affect the extraction efficiency of PFCAs because the existing forms of PFCAs in samples are different at different pH values. In this work, pH values were evaluated in a range of 2 to 10. The results in Figure 1C indicated that when the pH of the samples was too high or too low, the adsorption between the membrane and analytes was weakened. An optimal adsorption efficiency was observed when the samples were neutral or the pH was around 7.

#### 2.2.3. Optimization of Desorption Conditions

**Figure 2:**
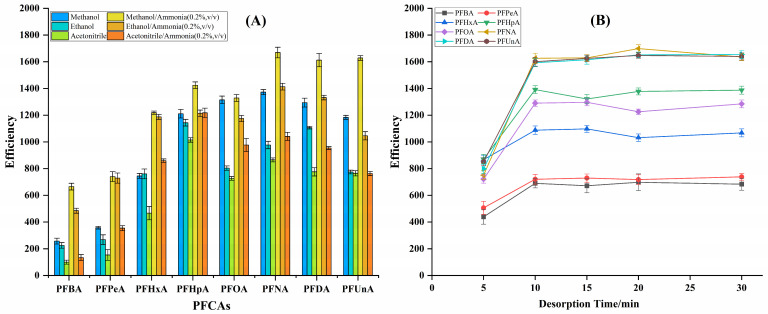
Optimization of the NCDs@HFM-SPME conditions for the desorption of PFCAs. The effects of (**A**) desorption solvent composition and (**B**) desorption time (from 0 to 30 min) on the extraction efficiency are evaluated. Optimization experiments were performed using a standard solution containing the eight target PFCAs, each at a concentration of 1.0 μg/L. Error bars represent the standard deviation of triplicate measurements.

Desorption solvent and desorption time were optimized to ensure complete analyte release from the NCDs@HFM. Methanol, ethanol, acetonitrile, and their mixtures with 0.2% (*v*/*v*) ammonia were evaluated as desorption solvents. As illustrated in Figure 2A, the 0.2% ammonia in methanol solution achieved the highest desorption recovery for all PFCAs and was therefore selected for all subsequent experiments. The effect of desorption time was evaluated from 5 to 30 min. As shown in Figure 2B, the desorption efficiency increased gradually with time up to 10 min. When the desorption time is extended from 10 to 15 min, the desorption efficiency for most anionic perfluorinated compounds remains constant. These findings indicate that 10 min is sufficient to achieve optimal desorption efficiency.

### 2.3. Investigation of Matrix Effects

Considering the potential interferences from common matrix compounds (such as long-chain fatty acids and other organic components), the evaluation of matrix effects is an important issue for validating the reliability of the proposed method. In this experiment, the matrix effect was quantitatively assessed using the blank water sample spiking method. Blank samples of representative matrices (tap water, river water, and industrial wastewater) were selected and spiked with the PFCA standard mixture to a final concentration of 1.0 ng/mL. The spiked samples were then processed through the complete NCDs@HFM-SPME procedure. For comparison, the corresponding standard solution at the identical concentration was handled according to the same extraction procedure. The ME for each analyte was calculated using the following equation:

$$ME\;\left(\%\right)=\;\left(\frac{A_{matrix}}{A_{pure}}-1\right)\times 100\%,$$

where *A_matrix_* and *A_pure_* represent the peak areas of the analyte obtained from the spiked matrix samples and the pure standard solution, respectively. As summarized in Table S1 (Supplementary Material), the ME values for all target PFCAs across the three water matrices ranged from −14.5% to +13.8%, indicating that acceptable matrix effects were observed by NCDs@HFM-SPME. These results demonstrated that the sample cleanup using the NCDs@HFM effectively removed most co-extracted matrix components, confirming the robustness and reliability of the developed method for PFCAs analysis in complicated environmental water samples.

### 2.4. Method Validation

In this work, the method validation was designed and performed in accordance with the International Council for Harmonisation (ICH) guideline Q2 (R2). Following the guidelines, the analytical characteristics of the proposed method were comprehensively validated in terms of linearity, sensitivity, accuracy, and precision under the optimum conditions. Good linearity was achieved for the eight PFCAs across their respective concentration ranges. The correlation coefficients (R^2^) for the calibration curves were greater than 0.995, confirming a strong linear relationship. Method sensitivity was assessed through the limits of detection (LODs) and quantification (LOQs), which were determined based on signal-to-noise (S/N = 3 for LOD and S/N = 10 for LOQ). As summarized in Table 1, the LODs and LOQs were in the ranges of 1.8–15 ng/L and 6–50 ng/L, respectively, demonstrating high sensitivity of the developed method. The accuracy of the method was evaluated by recovery experiments using standard addition at three concentration levels. Precision of the method was investigated as both intra-day reproducibility and inter-day reproducibility. As shown in Table 2, satisfactory recoveries were obtained, ranging from 70.6% to 122.5%, with relative standard deviations (RSDs) all below 13.2%. These results confirmed the good accuracy and repeatability of the proposed method for determining PFCAs in aqueous samples.

### 2.5. Practical Application in Environmental Water Samples

The proposed NCDs@HFM-SPME-LC-MS/MS method was applied in the detection of eight typical PFCAs in environmental water matrices. All real samples were processed following the optimized procedure outlined in Section 3.3. Quantification was performed using the external calibration curves established during method validation. The concentrations of the detected PFCAs in the different water samples are summarized in Table 3. Target PFCAs were detected at varying levels across different sample matrices. PFOA was the most frequently detected and predominant compound, with concentrations ranging from 0.32 to 6.1 μg/L, notably higher in industrial wastewater samples.

### 2.6. Methods Comparison

To critically evaluate the performance of the developed NCDs@HFM-SPME-LC-MS/MS method, a comparative analysis with other reported procedures was conducted. The key metrics of each method were summarized in Table 4. The comparison illustrated that the proposed method exhibited a competitive combination of high sensitivity, good accuracy and precision in the trace-level monitoring of PFCAs in environmental waters.

## 3. Materials and Methods

### 3.1. Materials and Chemicals

**Figure 3:**
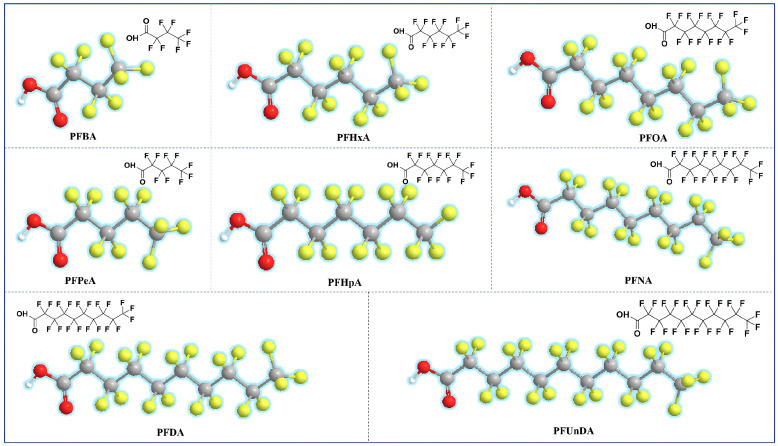
The structures of typical perfluoroalkyl carboxylates investigated in the proposed work.

Perfluoroalkyl carboxylates referenced materials, including PFBA, PFPeA, PFHxA, PFHpA, PFOA, PFNA, PFDA and PFUnDA, were purchased from Aladdin Chemistry Co., Ltd. (Shanghai, China). All of the eight standards are depicted in detail in Figure 3. The mixed standard stock solution of 1.0 μg/mL was prepared by dissolving each PFCA in methanol, respectively. Working standard solutions were prepared by appropriately diluting the stock solutions with methanol. All the solutions were stored at 4 °C in the refrigerator. Anhydrous citric acid (CA, 99.5%), polyethyleneimine (PEI, 98%, Mw = 1200), aqueous ammonia (28%), potassium permanganate (KMnO_4_, 99.5%) and sodium hydroxide (NaOH, 97%), Trimesoyl chloride (TMC, 98%) were purchased from Aladdin Chemistry Co., Ltd. (Shanghai, China). Methanol, acetonitrile and ethanol of HPLC-grade were purchased from Tedia Company (Fairfield, OH, USA). Ultra-pure water was obtained from a Milli-Q purification system (Millipore Co., Bedford, MA, USA). Hollow fiber membrane (800 μm i.d.) with a wall thickness of approximately 200 μm was supplied by Focused Photonics Incorporated Company (Hangzhou, China). The HFM is composed of polyacrylonitrile (PAN), featuring a nominal porosity of ~40% and an average pore size of 0.2 µm.

Environmental water samples were collected randomly from an industrial area (e.g., dyestuff industrial wastewater) and a living district in Zhejiang Province of China. All samples were filtered with a 0.22 μm nylon filter membrane to remove the suspended substance and stored at 4 °C for further use.

### 3.2. Preparation of NCDs and NCDs@HFM

The synthesis of NCDs used a modified bottom-up strategy. To be specific, 4.0 g of anhydrous CA and 3.2 mL of PEI were employed as carbon source precursor and nitrogen source precursor. The precursors were homogeneously mixed in 20 mL of deionized water and subjected to hydrothermal carbonization in a 50 mL high-pressure autoclave at 180 °C for 4 h. After the thermal decomposition process, the resulting brownish yellow product was purified by a dialysis bag (molecular weight cutoff (MWCO): 3.5 kDa) to remove residual molecular precursors and small weight by-products. Afterwards, the NCDs solution was vacuum dried and stored at 4°C for future use.

**Figure 4:**
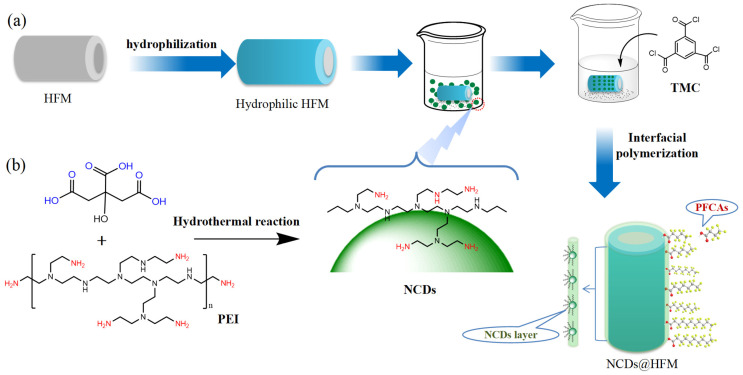
Schematic illustration of the fabrication strategy for the nitrogen-doped carbon quantum dots functionalized hollow fiber membrane (NCDs@HFM). (**a**) Stepwise procedure for the interfacial polymerization and functionalization of the hollow fiber membrane (HFM) with the NCDs to form the NCDs@HFM composite; (**b**) synthesis process of the nitrogen-doped carbon dots (NCDs) used in this work.

Subsequently, NCDs were incorporated with the hollow fiber membrane substrates to fabricate NCDs@HFM. Specifically, a series of homogeneous PEI/NCD dispersions was prepared by ultrasonically dispersing different amounts of NCDs (0, 10, 20, 30, and 50 mg) into 20 mL of an aqueous PEI solution (0.50 wt%) for 20 min. Each PEI/NCD dispersion served as the aqueous monomer solution. The membrane was prepared through hydrolysis in a 2.0 mol/L NaOH solution for 20 min. Subsequently, the membrane was immersed in the PEI/NCDs dispersion for 10 min. Then the membrane was transferred to another organic monomer solution, which consisted of 0.20 w/v% TMC in n-hexane and reacted for 5 min to immobilize the NCDs onto the hollow fiber membrane surface. Finally, the solution was discarded and the composite membrane was air-dried and stored at room temperature for future utilization. The whole protocol of the preparation was depicted in Figure 4.

### 3.3. SPME Procedure

The proposed SPME procedure involved two main steps. Firstly, the NCDs@HFM was fitted to a SPME module. The hollow fiber part was immersed into 10 mL aqueous sample solution. The extraction step lasted for 20 min with stirring (200 rpm). Then the module was removed from the sample and immersed in 1.0 mL of desorption solvent to release the target compounds. The desorption solution was then completely evaporated under a nitrogen stream at 40 °C. Finally, the residue was re-dissolved in 0.2 mL of pure methanol and ready for liquid chromatography-tandem mass spectrometry (LC-MS/MS) analysis.

Unlike many conventional SPME fibers that suffer from brittleness and limited lifespan, the NCDs@HFM offers a distinct advantage in durability. To ensure reusability, the NCDs@HFM was regenerated after each analysis. It was sequentially immersed with stirring in (i) a methanol/water solution (7:3, *v*/*v*) for 20 min to remove residual matrix components, followed by (ii) a NaOH solution for 15 min to re-activate the adsorption sites. The regenerated fiber was rinsed with water and dried before the next use.

### 3.4. Instrumentation and LC-MS/MS Analytical Conditions

The instrumental analysis of PFCAs was performed on a LC-MS/MS system (Thermo Fisher, Waltham, MA, USA). The chromatographic separation was conducted using a Thermo Scientific UltiMate 3000 system (Waltham, MA, USA). Detection was carried out with a Thermo Scientific TSQ Quantum Access MAX triple quadrupole mass spectrometer (Waltham, MA, USA). The stationary phase used in this system was an Acclaim-C18 column (150 mm× 2.1 mm, 2.2 μm particle size). The mobile phase consisted of ammonium acetate aqueous solution (5 mmol/L, Solution A) and methanol (Solution B). A gradient elution procedure was programmed to successfully separate the target molecules. The specific condition of gradient elution was as follows: 0.0–7.0 min = 70–10%A, 7.0–12.0 min = held at 10% A, 12.0–12.1 min = 10–70% and held to 15.0 min. The flow rate was kept at 0.3 mL/min. The temperature of the column oven was set at 40°C while the injection volume was 1 μL.

As for mass spectrometry, an electrospray ionization (ESI) source operating in negative ion mode was used. The detection was performed in the multiple reaction monitoring (MRM) mode. For each target PFCAs, the optimal MRM parameters, including precursor ions, product ions and collision energies, were comprehensively listed in Table S2 (Supplementary Material). Other parameters were described as follows: the drying gas (nitrogen) temperature was set to 200 °C at a flow rate of 11 L/min, while the nebulizer pressure was maintained at 35 psi. The sheath gas parameters included a temperature of 150 °C and a flow rate of 8 L/min. The capillary and nozzle voltages were optimized to 3500 V and 2000 V, respectively, to ensure efficient ionization and signal stability.

### 3.5. Method Validation

The method validation process was designed and performed in accordance with the International Council for Harmonisation (ICH) guideline Q2 (R2) on Validation of Analytical Procedures. Following the guideline, key parameters including linearity, sensitivity, accuracy and precision were determined. Linearity was established by calibration curves across a defined concentration range. Sensitivity of the developed method was evaluated according to the limits of detection (LODs) and the limits of quantification (LOQs), which were calculated by three times the signal-to-noise ratio (S/N = 3) and ten times the signal-to-noise ratio (S/N = 10), respectively. Accuracy and precision were verified due to the recoveries and relative standard deviations (RSDs). Furthermore, to certify the potential effects of a complicated environmental sample matrix, the assessment of matrix effects (ME) was conducted.

## 4. Conclusions

In summary, this study developed a highly efficient and robust analytical method based on a novel nitrogen-doped carbon dots functionalized hollow fiber membrane (NCDs@HFM) for solid-phase microextraction (SPME), coupled with LC-MS/MS, for the sensitive monitoring of perfluoroalkyl carboxylates in environmental waters. The incorporation of nitrogen-doped carbon dots onto the hollow fiber membrane introduced abundant surface functional groups and a tailored core–shell structure. This design significantly enhanced the extraction performance through synergistic mechanisms: the protonated nitrogen-containing groups provided strong electrostatic attraction for anionic PFCAs, while the specific surface chemistry and structure facilitated effective interactions with both short- and long-chain PFCAs. This established NCDs@HFM-SPME-LC-MS/MS method demonstrated excellent analytical performance. It achieved wide linear ranges, low detection limits at the ng/L level, and satisfactory accuracy and precision with good recoveries for spiked samples. The proposed method stands as a promising tool for routine monitoring and study of trace-level PFCAs contamination, contributing to improved environmental and human health risk assessment. Future research may focus on extending this method to on-site sample preparation and rapid screening of emerging contaminants by exploring its integration into automated and portable monitoring systems.

## Figures and Tables

**Table 1:** Linear ranges, correlation coefficients, LODs and LOQs of PFCAs.

PFCAs	Linear Range (ng/mL)	Correlation Coefficients	LOD (ng/L)	LOQ (ng/L)
PFBA	0.05–20	0.9967	15	50
PFPeA	0.02–20	0.9987	6	20
PFHxA	0.02–20	0.9953	6	20
PFHpA	0.02–20	0.9971	3	10
PFOA	0.02–20	0.9982	3	10
PFNA	0.02–20	0.9965	1.8	6
PFDA	0.02–20	0.9955	3	10
PFUnDA	0.02–20	0.9964	4.5	15

**Table 2:** Evaluation of accuracy and precision using spiked blank water samples.

PFCAs	Spiked Level (ng/mL)	Average Recovery (%)	Intra-Day RSD (%)	Inter-Day RSD (%)
PFBA	0.10	92.4	6.1	7.6
	0.50	94.8	10.3	12.7
	5.00	93.6	9.7	11.5
PFPeA	0.10	87.4	11.4	10.7
	0.50	100.8	5.7	9.4
	5.00	98.7	9.5	11.2
PFHxA	0.10	89.5	8.8	9.2
	0.50	93.9	7.1	7.3
	5.00	106.9	8.8	10.2
PFHpA	0.10	86.4	12.6	10.5
	0.50	70.6	9.7	13.2
	5.00	92.3	3.9	5.4
PFOA	0.10	105.7	7.9	9.6
	0.50	96.5	12.2	9.4
	5.00	96.3	8.2	10.3
PFNA	0.10	89.8	7.2	8.6
	0.50	92.3	5.1	7.3
	5.00	91.6	5.9	6.9
PFDA	0.10	110.6	8.1	6.5
	0.50	85.6	7.2	10.7
	5.00	115.3	6.0	7.4
PFUnDA	0.10	122.5	9.4	7.0
	0.50	117.8	8.3	8.6
	5.00	97.8	10.6	5.3

**Table 3:** Analytical results of the determination of PFCAs in real water samples using the proposed method (n = 3).

Sample	Concentration (μg/L)							
	PFBA	PFPeA	PFHxA	PFHpA	PFOA	PFNA	PFDA	PFUnDA
Industrial wastewater A	N.D. ^a^	0.10	0.08	N.D	3.1	N.D.	N.D.	N.D.
Industrial wastewater B	1.2	N.D.	N.D.	N.D.	6.1	N.D.	N.D.	N.D.
Industrial wastewater C	N.D.	N.D.	N.D.	N.D.	4.2	N.D.	N.D.	N.D.
Industrial wastewater D	1.7	N.D.	0.35	0.13	2.9	N.D.	N.D.	N.D.
Rain water	0.36	0.13	N.D.	0.25	0.88	N.D.	N.D.	N.D.
River water	0.6	N.D.	N.D.	0.18	0.32	N.D.	N.D.	N.D.
Tap water	N.D.	N.D.	N.D.	N.D.	N.D.	N.D.	N.D.	N.D.
Drinking water	N.D.	N.D.	N.D.	N.D.	N.D.	N.D.	N.D.	N.D.

^a^ N.D. means not detected (<LOD).

**Table 4:** Comparison between the proposed method and the reported methods in the literature.

Methods	Sample Matrix	Concentration Rate	LOD	Recovery	Reference
NCDs@HFM-SPME-LC-MS/MS	Environmental water	35–61	0.0018–0.015 μg/L	70.6–122.5%	This research
MBA/MMF-SPME-HPLC-MS/MS	Water and vegetable samples	/	0.00011–0.00086 μg/L	80.6–120%	[33]
PM/MME-SPME-HPLC-MS/MS	Fish and seafood	/	0.0062–1.5 μg/kg	80.2–119%	[34]
QuECHERS-LC-MS/MS	Aquatic samples	/	0.001–0.070 μg/kg	71.7–120%	[35]
FM-SPE-HPLC-MS/MS	milk	21.91–100.6	0.005–0.05 ng/L	81.31–128.07%	[36]

## Data Availability

Data is contained within the article. The original contributions presented in this study are included in the article. Further inquiries can be directed to the corresponding authors.

## Supplementary Materials

The following supporting information can be downloaded at: https://www.mdpi.com/article/10.3390/molecules31081255/s1 Figure S1: Chromatogram of PFCAs standard solution; Figure S2: Chromatogram of the representative environmental water sample; Table S1: Matrix effect values for target PFCAs in different water matrices; Table S2: MRM parameters in this work.
