# Supplementary material for: Development of Solid-Phase Microextraction with Carbon Dot-Functionalized Hollow Fiber Membrane for the Analysis of Perfluoroalkyl Carboxylates in Aqueous Samples
Source: Molecules. 2026 Apr 10;31(8):1255. doi: 10.3390/molecules31081255 (PMC13118403; doi:10.3390/molecules31081255)
Supplement: Supplementary file 1 [file molecules-31-01255-s001.zip › molecules-4063695-supplementary.pdf]

Table S1. Matrix effect values for target PFCAs in different water matrices

**PFCAs**	**Tap water (%)**	**River water (%)**	**Industrial wastewater (%)**
PFBA	+5.6	-3.6	-9.8
PFPeA	+7.6	+2.8	-4.5
PFHxA	+11.4	+10.3	+9.6
PFHpA	-10.8	-7.3	-14.5
PFOA	-9.4	+12.9	+6.6
PFNA	+2.7	-7.7	+13.8
PFDA	+4.2	+5.9	-6.2
PFUnDA	-3.1	+5.3	+1.5

Table S2. MRM parameters in this work

PFCAs	Precursor Ion	Product Ion	Collision Energy (eV)
PFBA	213.0	169.0	11
PFPeA	263.0	219.0	11
PFHxA	313.0	269.0*/119.0	12/21
PFHpA	363.0	319.0*/169.0	12/18
PFOA	413.1	369.0*/169.0	12/19
PFNA	463.0	419.0*/169.0	12/19
PFDA	513.0	469.0*/269.0	12/21
PFUnDA	563.0	519.0*/269.0	12/21

* means quantitative ions.

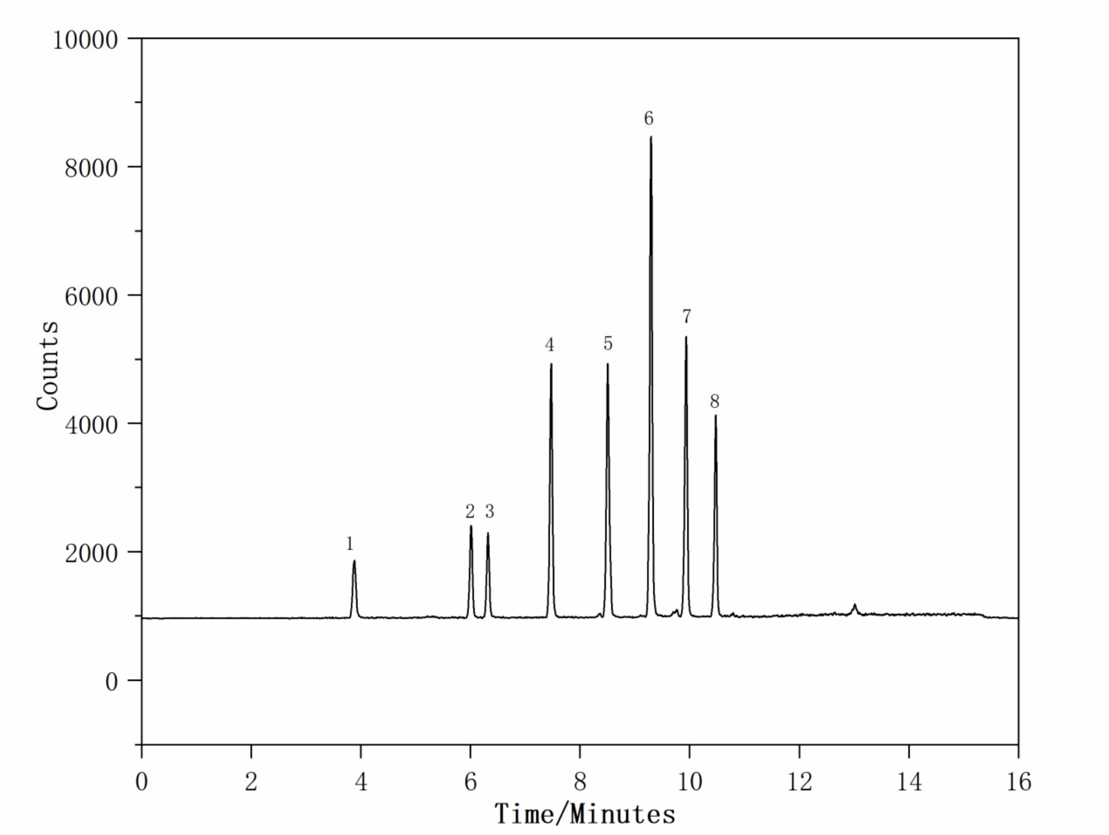

Figure S1. Chromatogram of PFCAs standard solution. Peaks: 1. PFBA, 2. PFPeA, 3. PFHxA, 4. PFHpA, 5. PFOA, 6. PFNA, 7. PFDA, and 8. PFUnDA.

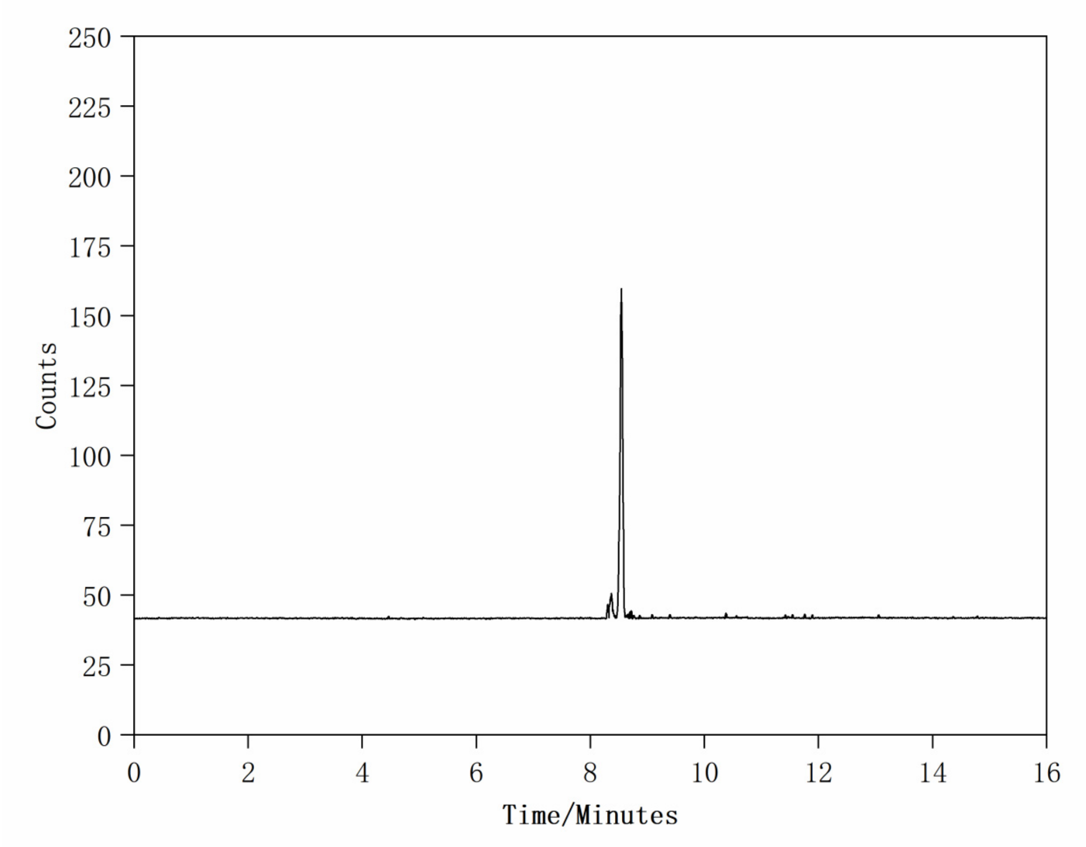

Figure S2. Chromatogram of the representative environmental water sample. The peak refers to PFOA.
